# Ileal Trichobezoar Presenting as Intestinal Obstruction and Peritonitis

**DOI:** 10.21699/ajcr.v8i2.549

**Published:** 2017-03-18

**Authors:** Kamal Nain Rattan, Vikas Yadav, Varun Yadav, Jasbir Singh

**Affiliations:** 1 Department of Pediatric Surgery, PGIMS, Rohtak, Haryana, India; 2 Department of General Surgery, MAMC, Agroha, Haryana, India; 3 Department of General Medicine, PGIMS Rohtak, Haryana, India; 4 Department of Pediatrics, PGIMS, Rohtak, Haryana, India

**Keywords:** Trichobezoars, Intestinal obstruction, Peritonitis

## Abstract

Trichobezoar is less common in boys. We are reporting a case of isolated ileal trichobezoars in a 4-year old boy causing intestinal obstruction and gut ischemia with perforation and peritonitis. The case was managed surgically with ileal resection and anastomosis. Postoperative period was uneventful.

## CASE REPORT

A four-year-old boy presented with abdominal distension, bilious vomiting and non-passage of flatus and stools for the last four days. He had abdominal pain for a month. Patient was adopted child with history of pica and trichotillomania. On general physical examination patient was thin built, malnourished with weight of only 10kg and poor orodental hygiene. On clinical examination, patient was dehydrated with pulse rate of 106/minute and blood pressure of 104/64 mm Hg. Abdomen was distended with signs of peritonitis. The laboratory analysis revealed hemoglobin of 9gm% with marked leucocytosis of 15000/mm3. Rest of the laboratory tests were within normal range. Plain abdominal radiograph showed multiple air-fluid levels. Ultrasound abdomen showed dilated gut loops. Laparotomy revealed significantly dilated and distended small bowel loops proximal to an impacted bezoar at distal ileum. There were two minute perforations and the gut wall ischemia at the site of impaction. The trichobezoar was extracted through enterotomy and ischemic ileal segment was resected, and end to end ileo-ileal anastomosis was performed (Fig.1). There were no bezoars in the stomach or any other part of bowel. The postoperative course was uneventful and the patient was referred to the psychiatry department for further management.

**Figure:**
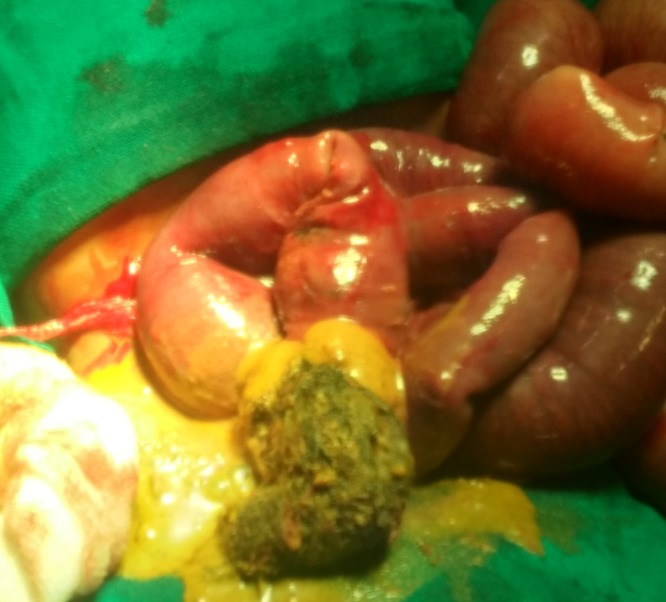
Figure 1: Showing enterotomy with trichobezoars extraction from terminal ileum

## DISCUSSION

Presentation of trichobezoars is generally late because it takes many months to form a considerable trichobezoar before an impaction can occur. Moreover, initially subacute intestinal obstruction and other vague signs and symptoms also causes delayed diagnosis in addition to low index of suspicion.[1-3] Most of the patients present with complications of trichobezoar such as intestinal obstruction, intestinal ischemia, and perforation peritonitis.[1-5] In the index case the child presented with acute intestinal obstruction and perforation at the impaction site leading to fecal peritonitis.

Laparotomy remains the treatment of choice for intestinal bezoars. In our patient during exploration small perforation was also found and gut wall was ischemic, so after enterostomy, resection and end to end anastomosis was also needed. Psychological support and psychiatric treatment is the corner stone for preventing recurrence.

## Footnotes

**Source of Support:** Nil

**Conflict of Interest:** None declared

